# The interaction effect of high social support and resilience on functional connectivity using seed-based resting-state assessed by 7-Tesla ultra-high field MRI

**DOI:** 10.3389/fpsyt.2024.1293514

**Published:** 2024-05-20

**Authors:** Nibal Khudeish, Shukti Ramkiran, Dominik Nießen, Dilsa Cemre Akkoc Altinok, Ravichandran Rajkumar, Jürgen Dammers, N. Jon Shah, Tanja Veselinovic, Irene Neuner

**Affiliations:** ^1^ Institute of Neuroscience and Medicine, Institute of Neuroscience and Medicine (INM-4), Forschungszentrum Jülich GmbH, Jülich, Germany; ^2^ Department of Psychiatry, Psychotherapy and Psychosomatics, RWTH Aachen University, Aachen, Germany; ^3^ Jülich Aachen Research Alliance - Brain (JARA – BRAIN) – Translational Medicine, Aachen, Germany; ^4^ Institute of Neuroscience and Medicine, Institute of Neuroscience and Medicine (INM-11), Forschungszentrum Jülich GmbH, Jülich, Germany; ^5^ Department of Neurology, Rheinisch-Westfälische Technische Hochschule Aachen (RWTH) Aachen University, Aachen, Germany

**Keywords:** resilience, social support, UHF fMRI, healthy population, cognitive processing, emotion regulation, mental health, functional connectivity

## Abstract

Recent resilience research has increasingly emphasized the importance of focusing on investigating the protective factors in mentally healthy populations, complementing the traditional focus on psychopathology. Social support has emerged as a crucial element within the complex interplay of individual and socio-environmental factors that shape resilience. However, the neural underpinnings of the relationship between social support and resilience, particularly in healthy subjects, remain largely unexplored. With advances in neuroimaging techniques, such as ultra-high field MRI at 7T and beyond, researchers can more effectively investigate the neural mechanisms underlying these factors. Thus, our study employed ultra-high field rs-fMRI to explore how social support moderates the relationship between psychological resilience and functional connectivity in a healthy cohort. We hypothesized that enhanced social support would amplify resilience-associated connectivity within neural circuits essential for emotional regulation, cognitive processing, and adaptive problem-solving, signifying a synergistic interaction where strong social networks bolster the neural underpinnings of resilience. (n = 30). Through seed-based functional connectivity analyses and interaction analysis, we aimed to uncover the neural correlates at the interplay of social support and resilience. Our findings indicate that perceived social support significantly (p<0.001) alters functional connectivity in the right and left FP, PCC, and left hippocampus, affirming the pivotal roles of these regions in the brain’s resilience network. Moreover, we identified significant moderation effects of social support across various brain regions, each showing unique connectivity patterns. Specifically, the right FP demonstrated a significant interaction effect where high social support levels were linked to increased connectivity with regions involved in socio-cognitive processing, while low social support showed opposite effects. Similar patterns by social support levels were observed in the left FP, with connectivity changes in clusters associated with emotional regulation and cognitive functions. The PCC’s connectivity was distinctly influenced by support levels, elucidating its role in emotional and social cognition. Interestingly, the connectivity of the left hippocampus was not significantly impacted by social support levels, indicating a unique pattern within this region. These insights highlight the importance of high social support levels in enhancing the neural foundations of resilience and fostering adaptive neurological responses to environmental challenges.

## 1 Introduction

Understanding the protective factors that contribute to mental health resilience is a critical area of research, (1). Recent studies have shifted the focus towards mentally healthy populations (2, 3), moving beyond the traditional emphasis on trauma-induced resilience (4, 5). This shift highlights the necessity of exploring general protective factors in resilience, applicable across various contexts, not just specific traumatic experiences (1, 6). This broader approach allows for a more inclusive understanding of resilience factors that are relevant to a wider demographic.

Traditionally, resilience has been defined as an individual’s intrinsic ability to successfully navigate adversity (7). However, this definition fails to capture the complexity of the construct. it is not merely an individual trait but a dynamic process that unfolds at the intersection of multiple factors — genetic, epigenetic, developmental, and neurobiological — and is further shaped by broader socio-environmental contexts, including familial, cultural, and economic dimensions (7–11).

Given the complex interplay of individual and socio-environmental factors in shaping resilience, the role of social support has emerged as particularly salient in contemporary research (9). Numerous studies underscore the beneficial impact of social support in bolstering resilience (12, 13) and promoting mental health (13–16). Social support, fundamentally, represents the network available in times of need, providing emotional, physical, and financial assistance (www.cancer.gov) (12). Crucially, the delineation between structural facets (like network breadth and interaction frequency) and functional elements (encompassing perceived emotional and tangible support) of social support is paramount. It is worth noting that relationship quality, a facet of the functional dimension, often stands out as a key health predictor (17–19).

Exploring the neural underpinnings of the relationship between social support and resilience necessitates a nuanced understanding of findings from both task-based and resting-state fMRI studies. While resting-state fMRI can illuminate baseline brain connectivity patterns associated with resilience and social support, task-based fMRI could be instrumental in examining how resilient individuals handle challenges or stressors, thereby highlighting brain functionality during adversity. Holz et al. (20) provide a comprehensive review of the current literature on the neural mechanisms of resilience, particularly emphasizing the role of supportive social environments in modulating neural substrates involved in stress and emotion processing. Eisenberger (21) reviews task-based fMRI studies, focusing on the neural basis of receiving and giving social support, revealing activation in safety-related neural regions and reduced threat responses. Sato et al. (22) complement this with their resting-state fMRI findings, showing a link between elevated social support and decreased amygdala activation, indicating a potential neural pathway through which social support might mitigate stress responses. Alongside these neuroimaging insights, the behavioral research by Li et al. (23) delves into the impact of social support sources and resilience on mental health across various age groups during the COVID-19 pandemic. Their findings indicate that resilience positively predicts mental health, with social support serving as a buffer against the negative effects of low resilience. This pattern is consistent across age groups, underscoring the vital role of high social support in enhancing resilience and mental health. Collectively, these studies from both neuroimaging and behavioral perspectives reinforce the hypothesis that social support is integral to fostering resilience and promoting mental health.

Building on the exploration of the neural correlates of social support and resilience, we examine key neuroimaging studies that shed light on the mechanisms underlying resilience. Van Der Werff et al. (24) provide a comprehensive review of neuroimaging research, revealing how structural, resting-state, and task-related neuroimaging results capture the brain’s adaptive recovery following stress. Complementing this, the review by Bolsinger et al. (25) focuses on the neuroimaging correlates of resilience to traumatic events, detailing how structural changes and functional connectivity alterations occur in individuals with post-traumatic stress disorder PTSD, and how these changes relate to resilience. Roeckner et al. (26) and Swartz et al. (27) further add to this narrative by highlighting the correlations between specific neural networks and resilience, and the predictive power of amygdala reactivity for stress vulnerability, respectively. These combined insights from various neuroimaging studies enrich our understanding of the neural dynamics of resilience.

The advancements in neuroimaging technology, particularly resting-state fMRI and ultra-high field (UHF) MRI systems, further augment our capacity to delve into the neural aspects of resilience (28–31). Meyer-Lindenberg and Tost (32) have highlighted the significance of these modern tools in exploring the interplay of biological and environmental factors on brain function. Resting-state fMRI, as explored in the ‘Conceptualizing Psychological Resilience Through Resting-State Functional MRI in a Mentally Healthy Population’ review, illuminates the spontaneous brain activity related to resilience, demonstrating its complexity within mentally healthy populations (1). Furthermore, the introduction of UHF MRI systems, particularly at 7 Tesla, notably enhances spatial resolution and signal strength, as shown by van der Zwaag et al. (33) and Altinok et al. (34). This technological leap enables a more nuanced understanding of subtle brain activity variations, enriching our insights into the brain’s functionality in relation to resilience and social support.

Leveraging recent advancements in neuroimaging and anchored by the notion that high social support is integral to fostering resilience, this study employs ultra-high field resting-state fMRI (UHF rs-fMRI) to investigate the interplay between social support and resilience in healthy individuals. Our analysis focuses on key neural regions identified as central to resilience, including the frontal pole (FP), anterior cingulate cortex (ACC), posterior cingulate cortex (PCC), hippocampus, and amygdala, as highlighted in the reviews by Van Der Werff et al. (24) and Bolsinger et al. (25). Using a seed-to-voxel approach with a multiple regression model, we are keen to study the moderation role of high perceived social support on resilience and functional connectivity. We hypothesize that at higher levels of social support, the positive effect of resilience on seed-to-whole brain connectivity is amplified, particularly in brain regions related to emotion regulation, cognitive processing, and adaptive problem-solving. This suggests a synergistic interaction where the presence of strong social support enhances the neural correlates of resilience, facilitating adaptive responses to environmental demands and stressors. This study aims to reveal new neural pathways at the crossroads of social support and resilience, potentially guiding future resilience enhancement strategies in the general population.

## 2 Materials and methods

### 2.1 Participants

A total of 35 healthy volunteers were recruited in Aachen, Germany, for this study, which was part of a planned project to identify the neural underpinnings of resilience. All the participants were right-handed, native German speakers with no history of neurological or psychiatric disorders, as defined by the Diagnostic and Statistical Manual of Mental Disorders (DSM-V). We employed a rigorous screening procedure to ensure the absence of subclinical psychiatric diseases and trauma exposure. This was achieved using the Structured Clinical Interview for DSM-IV (SCID-I) to identify and exclude any subclinical psychiatric conditions. Additionally, we employed two short versions of the Early Trauma Inventory (ETI) - the ETI Trauma List (18 items) and the ETI Trauma Symptoms (23 items) - to thoroughly screen for and exclude any history of traumatic events and trauma-related symptoms in our participants. This comprehensive screening process was pivotal in establishing a cohort of healthy volunteers devoid of confounding psychiatric or traumatic histories.

The Edinburgh Handedness Inventory was used to assess the participants’ right-handedness (35). A total of 30 participants were included in the final analysis. The mean age of participants (n = 30) was 29 years, SD 9.06 (range 19–49). The mean age of male participants (n = 13) was 29.4 years, SD 8.04, and that of female participants (n = 17) was 28.8 years, SD 10.02. The study was approved by the Ethics Committee of the Medical Faculty of RWTH Aachen University, and all participants provided written informed consent. The research was carried out following the Helsinki Declaration.

### 2.2 Psychological questionnaires

#### 2.2.1 Resilience scale

Psychological resilience was assessed using the German version of the Resilience Scale (RS-25) (36). The RS comprises 25 items rated on a seven-point Likert scale from 1 (strongly disagree) to 7 (strongly agree). The total RS-25 score ranged from 25 to 175, with higher scores reflecting greater resilience (37). The adequate internal consistency and empirical evidence for the reliability and validity of the German version of the resilience scale have been previously demonstrated by Wagnild and Young (37). The total score of the RS-25 should be used in our analysis (36).

#### 2.2.2 Social support scale (F-SozU)

The German version of the Social Support Scale, F-SozU was used to assess perceived social support. The scale has a total of 14 statements about social contacts, for which individuals indicate their level of agreement on a five-point Likert scale from 1 - “strongly disagree” to 5 - “strongly agree” (e.g., “I have friends/relatives who can also listen well from time to time, who are also good at listening when I want to talk”). In terms of content, the statements refer to the areas of emotional support (being liked and accepted by others; being able to share feelings; experiencing sympathy), practical support (being able to get practical help with everyday problems, for example, borrowing something, receiving practical advice, being relieved of tasks), and social integration (belonging to a circle of friends; undertaking joint activities; knowing people with similar interests). The higher scores indicate better social support. The German version of the F-SozU has been previously validated and is considered suitable for research (38).

### 2.3 Multicollinearity assessment

Demographic data were analyzed using SPSS, version 20 (IBM SPSS Statistics 20). To evaluate the potential impact of multicollinearity, the Variance Inflation Factor (VIF) was examined after regressing both independent variables: resilience and social support, separately with age and gender as covariates. As a result of this regression, R-square values of 0.524 and 0.334 were obtained for resilience and social support, respectively. Correspondingly, VIF values of 2.10 for resilience and 1.50 for social support were calculated. Generally, a VIF value less than 4 is considered moderate collinearity (39). Given the relatively low VIF scores in our study, the interaction in our regression model should not be significantly affected by multicollinearity.

### 2.4 MRI data acquisition

MRI data acquisition was performed at Forschungszentrum Juelich using a 7T Magnetom Terra scanner (Siemens Healthineers, Erlangen, Germany) equipped with a 1Tx/32Rx Head Coil 7T Clinical (Nova Medical, Wilmongton, MA, USA). The structural and functional data were acquired in a single session.

For structural MRI, anatomical images were obtained with a T1 weighted MP2RAGE sequence within a scan time of 9:15 minutes. The image matrix was set to 240 × 256, achieving a 0.75 mm^3^ isotropic resolution in 192 sagittal slices. A short echo time (TE) of 2.27 ms and a long repetition time (TR) of 4500 ms were used. T1 weighting was acquired with an inversion time (TI) of 1000 ms. The signal-to-noise ratio (SNR) was optimized using a flip angle of 4°.

For functional imaging, resting-state fMRI data were obtained using echo-planar imaging (EPI) with echo and repetition time, TE/TR, of 25 ms/2200 ms. A total of 273 fMRI volumes were acquired within a 10.05 min acquisition time with 36 slices and a slice thickness of 3.1 mm. The image matrix size was 64 x 64, and the FOV was 200 x 200 mm^2^, resulting in a 3.1 mm isotropic resolution. In our protocol, participants were instructed to keep their eyes closed. This approach was chosen to reduce visual input, thereby minimizing potential confounds in neural activity related to visual processing. To ensure participants remained awake, we conducted brief verbal checks both before and after the resting-state measurements. This eyes-closed method aligns with our broader multimodal research approach, where we often integrate simultaneous electrophysiological recordings, such as EEG or evoked potentials. This standardized approach ensures consistency and comparability across our neuroimaging and electrophysiological datasets.

### 2.5 MRI data analysis

#### 2.5.1 Image processing

The rs-fMRI data underwent analysis using the CONN toolbox (40) (v.22.a) (41) default pipelines, supported by SPM 12 (https://www.fil.ion.ucl.ac.uk/spm/software/spm12/) (42) and implemented in MATLAB R2023a. The preprocessing involved several stages (43). Firstly, Functional data were realigned using the SPM realign & unwarp procedure (44), where all scans were coregistered to a reference image (first scan of the first session) using a least squares approach and a 6-parameter (rigid body) transformation (45), and resampled using b-spline interpolation to correct for motion and magnetic susceptibility interactions. Temporal misalignment between different slices of the functional data (acquired in interleaved Siemens order) was corrected following the SPM slice-timing correction (STC) procedure (46, 47), using sinc temporal interpolation to resample each slice BOLD time-series to a common mid-acquisition time. Potential outlier scans were identified using ART as acquisitions with framewise displacement above 0.9 mm or global BOLD signal changes above five standard deviations (48, 49), and a reference BOLD image was computed for each subject by averaging all scans excluding outliers. The functional and anatomical data were then normalized into the standard MNI space and compartmentalized into grey matter, white matter, and CSF using the SPM unified segmentation and normalization strategy (50, 51). Both functional and anatomical data were resampled to a default 180x216x180mm bounding box, with 2mm isotropic voxels for functional data and 1mm for anatomical data, using 4th-order spline interpolation (49, 52). Last, functional data were smoothed using spatial convolution with a Gaussian kernel of 8 mm full-width half maximum (FWHM).

Further refinement was done using a standard denoising pipeline (43). This involved nuisance regression performed by removing noise components of white matter and cerebrospinal fluid (5 CompCor noise components), subject-motion parameters, outlier scans (scrubbing), and the effect of rest (53). Temporal band-pass filtering was applied with a frequency range of 0.008-0.09 Hz, and linear detrending was conducted to remove low-frequency drifts. The bandpass frequency filtering for the BOLD timeseries (54) was set between 0.008 Hz and 0.09 Hz. The CompCor (55, 56) noise components present in the white matter and CSF were deduced by averaging the BOLD signal and factoring in the major components orthogonal to the average BOLD signal, motion parameters, and outlier scans per participant’s eroded segmentation masks. Participants with over 50% of their volume lost during scrubbing, owing to significant head movement during the rs-fMRI scan, were subsequently excluded from the later stages of the analysis.

#### 2.5.2 Seed-to-voxel functional connectivity analysis

In the first-level analysis, Seed-based connectivity maps (SBC) were estimated to identify the spatial patterns of functional connectivity associated with specified seed regions. Eight regions of interest (ROIs) from the Harvard-Oxford atlas were selected (57). The basis of selection was the literature proposing these regions as key elements of resilience (24, 25). These included the right and left FP, ACC, PCC, right and left hippocampus, and right and left amygdala. The strength of functional connectivity was represented using Fisher-transformed bivariate correlation coefficients derived from a weighted general linear model (weighted-GLM) (43). The relationship between their BOLD signal time series was modeled for each seed area and target voxel. To account for potential transient magnetization effects at each run’s onset, scans were weighted by a step function, which was then convolved with an SPM canonical hemodynamic response function and rectified.

For second-level seed-to-voxel analyses (Group-level analyses), a multivariate General Linear Model (GLM) was used (43). The specific GLM formulation used was *Y* = *β*0 ​+ *β*1​(Resilience) + *β*2​(Social Support) + *β*3​(Resilience × Social Support) + *β*4​(Gender) + *β*5​(Age) + *ϵ*.

To evaluate the potential impact of multicollinearity between the two independent variables on the model, the Variance Inflation Factor (VIF) was calculated, as detailed in the multicollinearity assessment section.

Although the model includes the main effects of resilience and social support, our primary focus was on the interaction term *β*3​(Resilience × Social Support). This focus reflects the main goal of our study, which is to determine whether social support moderates the relationship between resilience and functional connectivity (represented by *Y*) within our seed regions. A separate GLM was estimated for each voxel, incorporating first-level connectivity measures at these voxels as dependent variables and groups or other subject-level identifiers as independent variables. Voxel-level hypotheses were then examined using multivariate parametric statistics, factoring in random effects across subjects and sample covariance across multiple measurements. Inferences were made at the level of individual clusters, which comprise groups of contiguous voxels. These cluster-level inferences utilized parametric statistics grounded in Gaussian Random Field theory (43, 58). The results were thresholded using a dual criterion: a cluster-forming voxel-level p-value of < 0.001 and a familywise corrected p-FDR < 0.05 at the cluster size level (59). To this end, we employed a contrast vector, *C* = [0,0,0,1,0,0], to examine the interaction term while accounting for other covariates specifically. A significant result for this contrast would indicate that the effect of resilience on functional connectivity is moderated by social support. In our analysis, resilience and social support scores were standardized to z-scores, a method involving subtraction of the mean and division by the standard deviation for each variable. This standardization is crucial in regression analysis to minimize multicollinearity, especially when incorporating interaction terms, as highlighted by (60) in their work on multiple regression. Additionally, as recommended by (61), centering variables facilitates a clearer interpretation of interaction effects by setting the predictors to have a mean of zero.

#### 2.5.3 Resilience and social support interaction analysis

To enhance our understanding of how resilience interacts with social support to influence functional connectivity, we conducted an interaction analysis, utilizing MATLAB R2023a to ensure methodological consistency, particularly since our analyses were based on the CONN toolbox (40), a MATLAB-based framework. Participants were divided into ‘low’ and ‘high’ social support categories using a median split of the social support scores, a method chosen to address the skewed distribution of our dataset and to facilitate a clear dichotomous examination of the impact of social support levels on neural connections.

We generated interaction plots to graphically represent the influence of resilience on functional connectivity for the two distinct social support groups. This involved extracting functional connectivity values from significant clusters where the interaction between resilience and social support was significant, as determined by our regression analyses within the CONN toolbox.

The choice of a median split, allowed for straightforward group comparisons while ensuring sufficient sample sizes for each subgroup. This approach simplified the social support spectrum into two categories, enabling a focused analysis to elucidate specific interaction effects that define the relationship between social support, resilience, and brain functional connectivity. The interaction plots, created in MATLAB, serve as a visual testament to these effects, providing clear insights into the differential impacts observed across the grouped data.

## 3 Results

### 3.1 Sample characteristics

Out of 35 participants initially enrolled, five were excluded from the study: three due to excessive head movement, resulting in more than 50% volume removal during data scrubbing, and two owing to their left-handedness. This led to a final sample of 30 participants. The overall mean age was 29 years (SD = 9.06), with an age span from 19 to 49 years. Male participants (n = 13) had a mean age of 29.4 years (SD = 8.04), while females (n = 17) averaged 28.8 years (SD = 10.02).

### 3.2 Behavioral data

**Figure 1:**
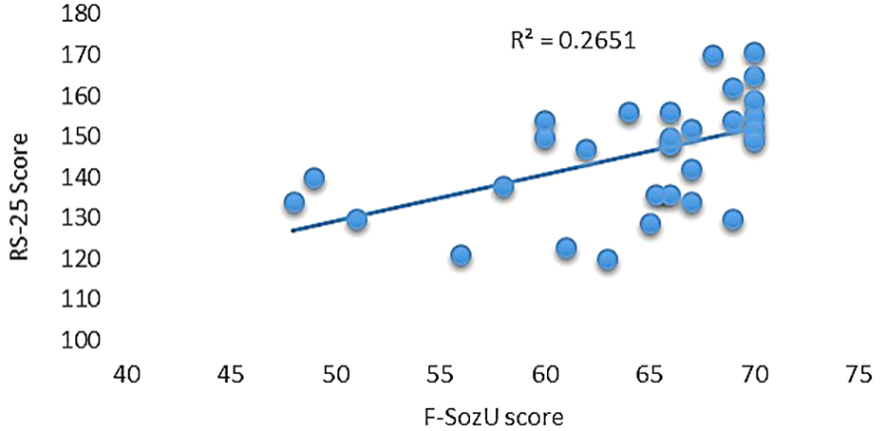
Correlation between resilience and social support scales.

Utilizing the RS-25 scale, participants demonstrated a moderate level of resilience, evidenced by an average score of 145.43 (SD = 14.07) and an observed range between 120 and 171. Furthermore, participants reported high levels of perceived social support as measured by the F-SozU scale, posting an average score of 64.08 (SD = 6.92) and scores spanning from 48 to 70. Notably, a significant positive correlation was observed between the two scales, yielding a correlation coefficient of 0.514 and accounting for 26.51% of shared variance (R^2 = ^0.2651, p = 0.003; see **Figure 1**).

### 3.3 Seed-based functional connectivity

In the seed-based functional connectivity analyses, significant alterations in connectivity influenced by the interaction between resilience and perceived social support were observed within four of our selected seed regions. This pattern underscores the specific moderating role of social support on the neural correlates of resilience (**Table 1**).

**Table 1:** Seed-based functional connectivity analysis results (resilience x social support).

Region of interest	Cluster #	MNI coordinates (x,y,z)	Cluster size (mm^3^)	Brain regions	p-unc	p-FDR	T-value	Effect size
FP-r	1	-44 -64 + 42	2,832	Lateral Occipital Cortex, superior division Left Angular Gyrus Left	0.000063	0.000063	4.84	0.24
FP-l	4	-58 -32 + 14 +14 + 38 + 44 -40 -72 + 46 +40 -60 + 44	984 1,128 992 872	Planum Temporale Left Parietal Operculum Cortex Left Superior Frontal Gyrus Right Frontal Pole Right Lateral Occipital Cortex, superior division Left Lateral Occipital Cortex, superior division Riht Angular Gyrus Right	0.000125 0.000028 0.000004 0.000190	0.000167 0.000055 0.000018 0.000190	-4.75 5.16 5.89 4.40	-0.23 0.22 0.19 0.18
PCC	1	-36 -74 -36	3,456	Cerebellum Crus2 Left Cerebellum Crus1 Left	0.000000	0.000000	6.88	0.16
Hippocampus-l	1	-08 + 44 + 22	864	Paracingulate Gyrus Left	0.000011	0.000011	5.54	0.14

MNI coordinates (x, y, z) represent peaks within a cluster. Cluster size corresponds to the spatial extent (i.e., volume (mm3)). Multiple comparisons were corrected using family-wise error correction at the cluster level.

In **Table 1**, the blue color represents decreased functional connectivity, while the red color represents increased functional connectivity.

**Figure 2:**
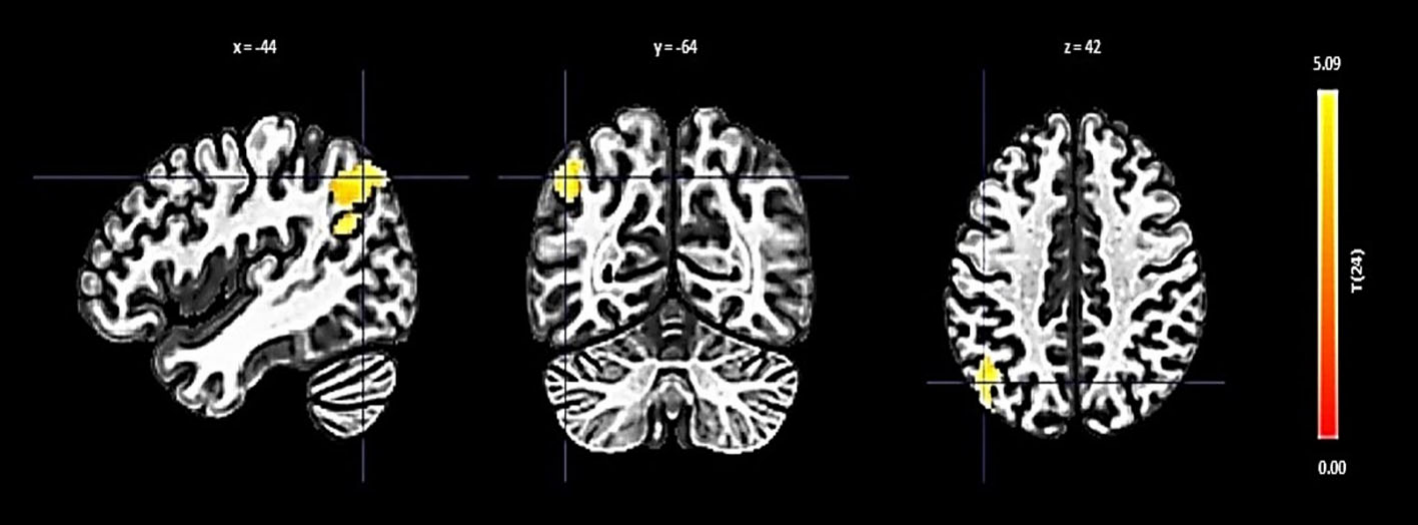
Seed-based functional connectivity analysis results in right FP, controlling for age and gender. Significance is indicated by peak voxel t-statistics on color bars, with cluster significance set at p-FDR < 0.05 and a voxel-level threshold of p < 0.001. The Right FP shows increased connectivity with a 354-voxel cluster, notably the Left Lateral Occipital Cortex (superior division) and Left Angular Gyrus.

The right FP displayed increased connectivity with a cluster incorporating the left lateral occipital cortex and left angular gyrus. This significant change was denoted by a size of 354 voxels, a t-value of 4.84, and a p-FDR of 0.000063 (**Figure 2**).

**Figure 3:**
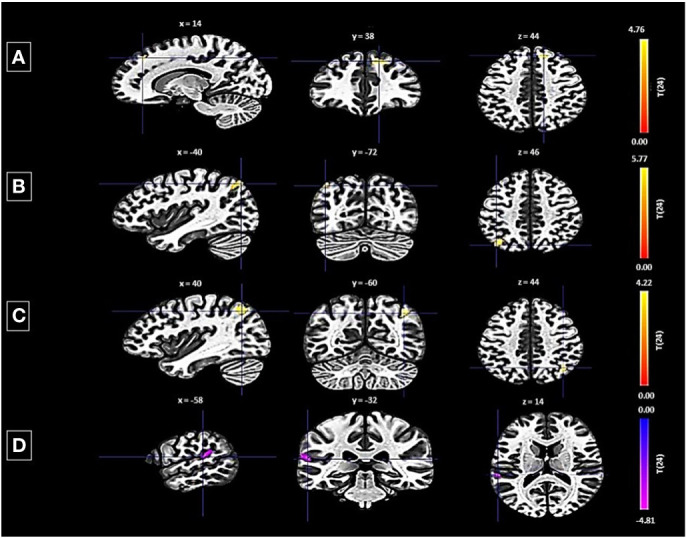
Seed-based functional connectivity analysis results in Left FP, controlling for age and gender. Significance is indicated by peak voxel t-statistics on color bars, with cluster significance set at p-FDR < 0.05 and a voxel-level threshold of p < 0.001. The left FP exhibits increased connectivity with three clusters: **(A)** Superior Frontal Gyrus Right and FP Right (141 voxels), **(B)** Right Lateral Occipital Cortex (superior division) and Right Angular Gyrus (109 voxels), and **(C)** Left Lateral Occipital Cortex (superior division) with 124 voxels. While exhibits decreased connectivity with a 123-voxel cluster, including the Planum Temporale Left and Parietal Operculum Cortex Left **(D)**.

Conversely, the left FP revealed both decreased and increased connectivity patterns with different clusters: a decrease was noted with a cluster encompassing the left planum temporale and parietal operculum cortex, shown by 123 voxels, a t-value of -4.75, and a p-FDR of 0.000167 (**Figure 3**). Enhanced connectivity was observed with the right superior frontal gyrus and right FP (size: 141 voxels, t-value: 5.16, p-FDR: 0.000055), the left lateral occipital cortex (size: 124 voxels, t-value: 5.89, p-FDR: 0.000018), and the right lateral occipital cortex and right angular gyrus (size: 109 voxels, t-value: 4.40, p-FDR: 0.000190).

**Figure 4:**
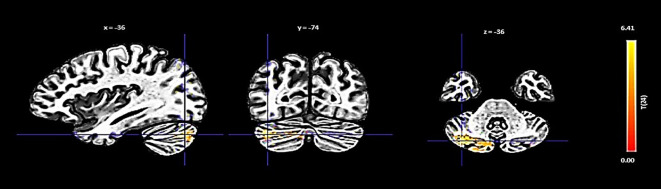
Seed-based functional connectivity analysis results in the PCC, controlling for age and gender. Significance is indicated by peak voxel t-statistics on color bars, with cluster significance set at p-FDR < 0.05 and a voxel-level threshold of p < 0.001. The PCC reveals increased connectivity within a 432-voxel cluster, with the Cerebellum Crus2 Left and Cerebellum Crus1 Left.

The PCC showed an increase in functional connectivity with a cluster encompassing the left cerebellum Crus 2 and Crus 1, denoted by a cluster size of 432 voxels, a t-value of 6.88, and a p-FDR of < 0.000001 (**Figure 4**).

**Figure 5:**
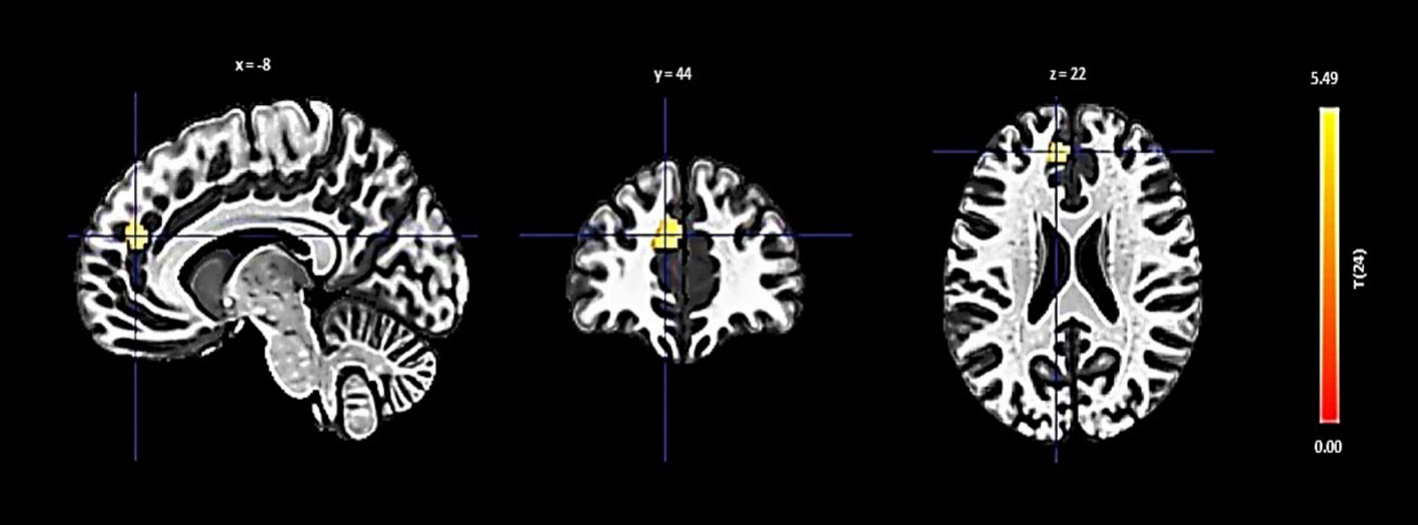
Seed-based functional connectivity analysis results in Left Hippocampus, controlling for age and gender. Significance is denoted by peak voxel t-statistics on color bars, with cluster significance set at p-FDR < 0.05 and a voxel-level threshold of p < 0.001. The Left Hippocampus demonstrates increased connectivity in a 108-voxel cluster, prominently with the Paracingulate Gyrus Left.

Lastly, the left hippocampus showed an increase in connectivity with a cluster including the left paracingulate gyrus, presented with a size of 108 voxels, a t-value of 5.54, and a p-FDR of 0.000011 (**Figure 5**).

### 3.4 Interaction effects of resilience and social support on functional connectivity

In our interaction analysis, we identified significant moderation effects of social support on the relationship between resilience and functional connectivity across various brain regions, each showing unique connectivity patterns.

**Figure 6:**
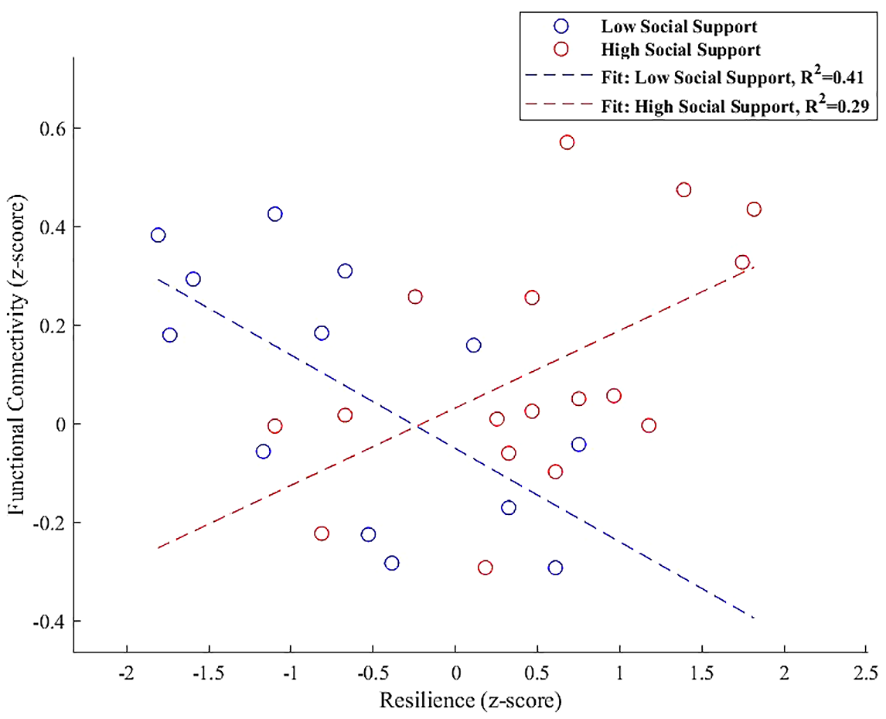
Interaction plot demonstrating the moderation effect of social support on the association between resilience and functional connectivity between the right FP and a cluster including the left lateral occipital cortex and left angular gyrus. Individuals with high social support exhibit a positive association between resilience and functional connectivity (β = 0.157, p = 0.025). Conversely, those with low social support show a significant negative association within the same regions (β = -0.189, p = 0.018), underscoring the influence of social support on neural resilience mechanisms.

In the right FP, the interaction plot illustrated in **Figure 6** revealed that individuals with high perceived social support exhibited a positive association between resilience and functional connectivity within a cluster that includes the left lateral occipital cortex and left angular gyrus (β = 0.157, SE = 0.063, t = 2.498, p = 0.025). In contrast, those with low social support demonstrated a significant negative association in the same region (β = -0.189, SE = 0.069, t = -2.765, p = 0.018), indicating that the level of social support significantly modulates the relationship between resilience and functional connectivity in the right FP.

**Figure 7:**
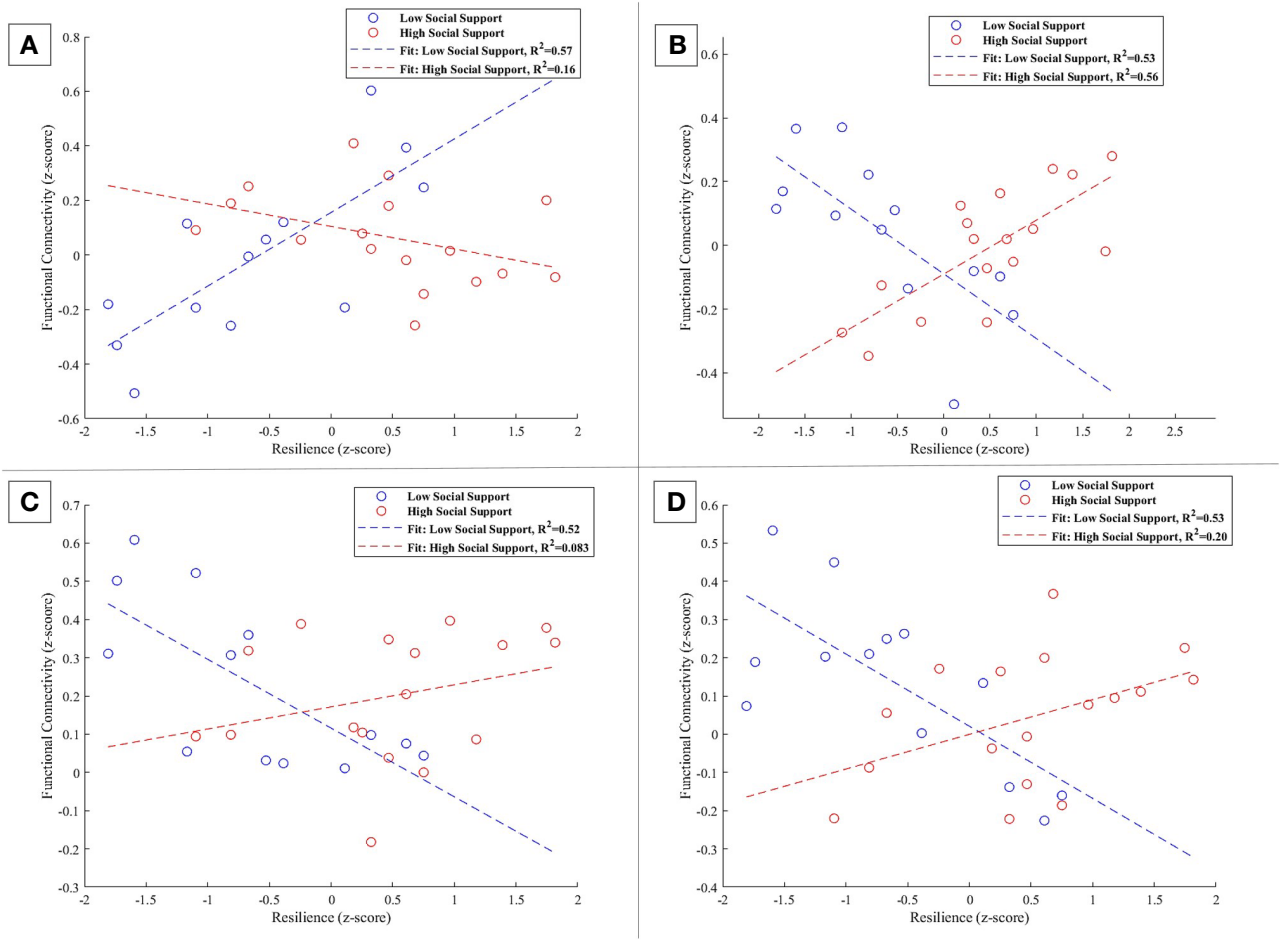
**(A-D)** Interaction plots demonstrating the moderation effect of social support on the functional connectivity associated with the left FP. In **(A)** low social support correlates with a statistically significant decrease in connectivity to the left planum temporale and parietal operculum cortex (β = -0.261, p = 0.003), whereas high social support is linked to an insubstantial positive effect (p = 0.243). **(B)** indicates a significant negative relationship with the superior frontal gyrus right and FP right in participants with low social support (β = -0.203, p = 0.0046), in contrast to a significant positive relationship in the high support cohort (β = 0.169, p = 0.0005). **(C)** demonstrates a substantial reduction in connectivity with the lateral occipital cortex, superior division left for those with low social support (β = -0.180, p = 0.0057), with no notable change for those with high support. Finally, **(D)** suggests a significant negative association for the low social support group with the lateral occipital cortex, superior division right, and angular gyrus right (β = -0.189, p = 0.0050), and an unsubstantial positive association for the high social support group (p = 0.0714).

In the interaction analysis of the left FP, we observed distinct modulation patterns of functional connectivity influenced by social support levels. The connectivity between the left FP and the left planum temporale and parietal operculum cortex exhibited a significant reduction in the low social support group (β = -0.261, SE = 0.074, t = -3.527, p = 0.003). Conversely, for the high social support group, a non-significant positive trend was noted in this pathway (β = 0.101, SE = 0.083, t = 1.218, p = 0.243), illustrated in **Figure 7A**.

Further analysis identified that the functional connectivity between the left FP and the cluster combining the superior frontal gyrus right and the FP right demonstrated a significant negative relationship for individuals with low social support (β = -0.203, SE = 0.057, t = -3.549, p = 0.0046). In contrast, a significant positive relationship was observed for individuals with high social support (β = 0.169, SE = 0.038, t = 4.413, p = 0.0005), as shown in **Figure 7B**.

For the connection involving the left FP and the lateral occipital cortex, superior division left, a significant negative correlation was reported for the low social support group (β = -0.180, SE = 0.053, t = -3.423, p = 0.0057), which is detailed in Figure BB. The high social support group’s connectivity in this region did not exhibit a significant change, as demonstrated in **Figure 7C**.

Additionally, the connectivity between the left FP and the region that includes the lateral occipital cortex, superior division right, and the angular gyrus right showed a significant negative correlation in the low social support group (β = -0.189, SE = 0.054, t = -3.495, p = 0.0050). A non-significant positive trend for the high social support group ((β = 0.091, SE = 0.047, t = 1.940, p = 00714) in this connectivity was illustrated in **Figure 7D**, underscoring the complex influence of social support levels on these neural interactions.

**Figure 8:**
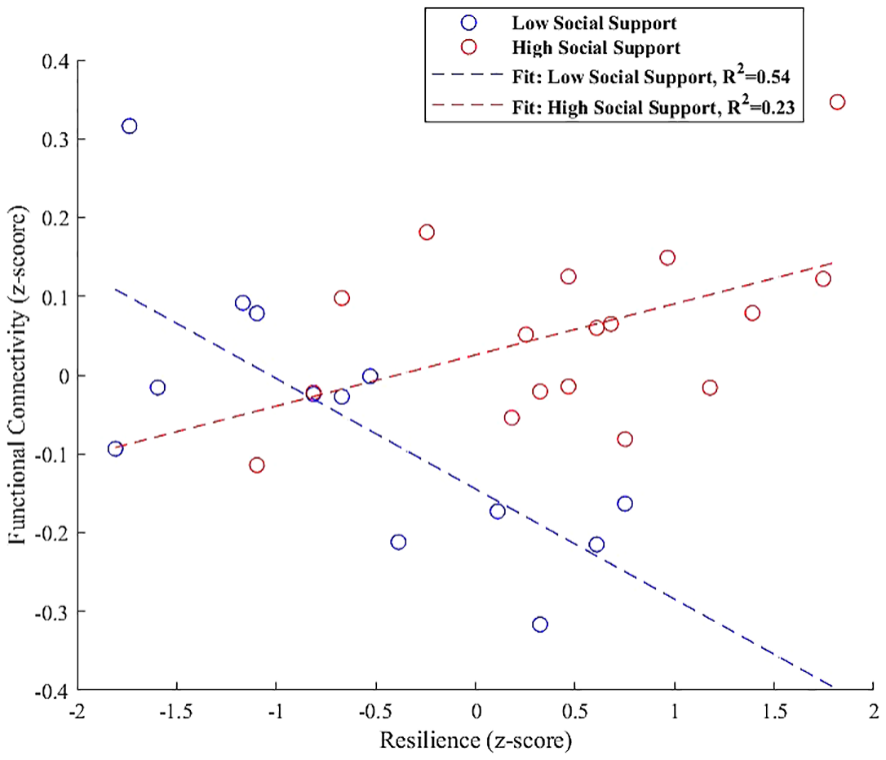
Interaction plot highlighting the moderation effect of social support on functional connectivity between the PCC and a cluster encompassing the left cerebellum Crus 1 and Crus 2. Low social support is associated with a significant negative effect on connectivity (β = -0.140, p = 0.0042), while high social support exhibits a positive, although weaker, association (β = 0.065, p = 0.049).

For the PCC, Increased connectivity with a cluster that includes the left cerebellum Crus 1 and Crus 2 was depicted in **Figure 8**, with a notable negative interaction effect for low social support (β = -0.140, SE = 0.039, t = -3.596, p = 0.0042) and a positive but weaker association for high social support (β = 0.065, SE = 0.030, t = 2.140, p = 0.049).

**Figure 9:**
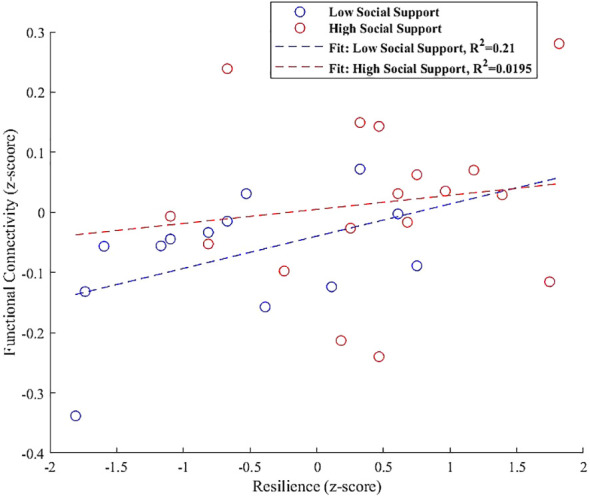
Interaction plot illustrating the moderation effect of social support levels on the relationship between resilience and functional connectivity in the left hippocampus, specifically with the left paracingulate gyrus. Trends indicate a slight, non-significant positive association for both low (β = 0.054, p = 0.120) and high social support groups (β = 0.023, p = 0.593), suggesting that the influence of social support on this connectivity is not pronounced in the current sample.

Finally, the interaction plot for the left hippocampus, shown in **Figure 9**, indicated increased connectivity with the left paracingulate gyrus. However, this did not significantly vary with social support levels, showing non-significant trends for both low (β = 0.054, SE = 0.031, t = 1.683, p = 0.120) and high social support groups (β = 0.023, SE = 0.043, t = 0.546, p = 0.593).

## 4 Discussion

Numerous studies have underscored the beneficial impact of high social support on psychological resilience, illustrating its positive effects across various populations. However, the intricate neural mechanisms that underpin this relationship, particularly in healthy individuals, remain less explored. Our study aimed to bridge this knowledge gap by exploring the moderating role of high perceived social support in shaping the interplay between resilience and resting-state functional connectivity. Building on the notion that high perceived social support is integral to fostering resilience, we hypothesized that high perceived social support significantly influences the functional connectivity within key brain regions associated with resilience. As hypothesized, we explored whether higher levels of social support amplify the positive effects of resilience on seed-to-whole brain connectivity, particularly in brain regions related to emotion regulation, cognitive processing, and adaptive problem-solving. This suggests a synergistic interaction where the presence of strong social support enhances the neural correlates of resilience, facilitating adaptive responses to environmental demands and stressors. To examine this hypothesis and the underlying neural pathways, we employed the CONN toolbox for a seed-based analysis, using a multivariate regression model to focus on eight seed regions: the right and left FP, ACC, PCC and the right and left hippocampus and amygdala. These regions have been identified in previous research as integral to resilience (24, 25).

Our findings revealed critical insights into how perceived social support modulates functional connectivity within essential brain regions. Specifically, social support notably influences connectivity in the right FP, PCC, and left hippocampus – regions pivotal for cognitive control, self-reflection, and stress adaptation. Furthermore, in the left FP, we observed a complex pattern of increased and decreased connectivity in different clusters, underscoring the multifaceted influence of social support on resilience-related neural mechanisms.

The interaction analysis further illuminated these dynamics, particularly showing that high social support correlates with positive connectivity between resilience and the right FP, involving key areas like the left lateral occipital cortex and left angular gyrus. In contrast, individuals with low social support showed a significant negative association in this region. For the PCC, a significant negative interaction effect with low social support was observed in its connectivity with the left cerebellum Crus 1 and Crus 2, whereas high social support demonstrated a positive, albeit weaker, relationship.

In the case of the left hippocampus, the interaction analysis indicated a relationship with the left paracingulate gyrus, yet this connectivity did not significantly vary with social support levels, presenting non-significant trends for both low and high support groups. These comprehensive findings, integrating results from both seed-to-voxel and interaction analyses, robustly affirm our hypothesis, emphasizing the significant moderating role of social support in the neural foundations of resilience.

Starting with our first seed region, the right FP we observed a notable increase in connectivity with a cluster encompassing the left lateral occipital cortex (superior division) and the left angular gyrus. This enhancement in connectivity underlines the crucial role of high perceived social support in strengthening neural connections within the right FP, particularly linking it to regions essential for visual processing and cognitive functionalities.

Our refined interaction analysis further clarifies the moderation role of perceived social support in this connectivity. At higher social support levels, an increase in functional connectivity is evident, correlating positively with resilience (β = 0.157, p = 0.025), underscoring the supportive framework’s potential to bolster adaptive neural processes within the FP. Conversely, under conditions of lower social support, we detect a decrease in connectivity, characterized by a significant negative association (β = -0.189, p = 0.018), thus highlighting the critical role of social support in modulating the neural dynamics of resilience within the FP.

The FP, a key part of the prefrontal cortex (PFC), plays a multifaceted role in cognitive and emotional processes, delineated into regions such as the lateral frontopolar area and the medial frontopolar area (62, 63). These areas are pivotal for tasks ranging from working memory and perception to the processing of emotional cues and understanding social interactions. The observed modulation of connectivity within the right FP, influenced by social support, aligns with these recognized functions, highlighting the FP’s role in orchestrating complex cognitive and emotional interactions.

Furthermore, the FP’s involvement in emotional cognition regulation and its significance in decision-making and executive control underscore its broader functional implications (64–66). The interaction analysis illustrates that high social support enhances the FP’s connectivity, potentially augmenting its capacity for value-driven decisions and goal-oriented behaviors (67, 68), particularly in socio-emotionally rich contexts.

Consistent with existing neuroimaging research, our findings confirm the FP’s connectivity with posterior visual areas and its collaboration with the lateral occipital cortex and angular gyrus, essential for visual and cognitive integration (69–72). This connectivity is crucial for the top-down regulation of emotions, engaging with other significant brain areas like the dorsolateral prefrontal cortex, ACC, and anterior insula (73, 74), and is reflective of the FP’s role in processing diverse emotional and cognitive information.

By demonstrating the FP’s enhanced connectivity under conditions of high social support, our findings contribute to the understanding of how social environments influence the neural correlates of resilience, offering insights into the brain’s adaptive mechanisms. This perspective is particularly relevant when considering the differential roles of the right and left prefrontal cortices in emotional processing (75–77) and the potential implications for stress-related adaptations and resilience, as suggested by research connecting frontal activity with PTSD outcomes (78).

Addressing the left FP, our observations resonate with the findings on the right FP, reinforcing the idea that resilience and social support synergistically enhance the brain’s capacity to integrate visual cues with social contexts. The left FP displayed a nuanced pattern of connectivity changes influenced by social support levels, as revealed in our interaction analysis.

Notably, the left FP demonstrated a decreased coupling with a cluster involving the operculum (PO) and planum temporale (PT) for individuals with lower social support levels (β = -0.261, p = 0.003). These regions are crucial for auditory processing and social cue integration, with the PT’s role in auditory speech processing being well-documented (79–84) and the PO’s involvement in sensory integration and processing (85, 86). Conversely, a non-significant positive trend in this connectivity was observed for the high social support group (β = 0.101, p = 0.243), suggesting a potential buffering effect of social support on auditory and social processing networks. Given the PT’s critical role in auditory processing and the PO’s involvement in sensory integration (79, 81), the observed reduction in connectivity may reflect a more focused cognitive state in individuals with lower social support, enabling better filtering of irrelevant sensory information, a function that is particularly vital considering the links between executive inhibition deficits and disorders like ADHD and PTSD (87, 88).

Transitioning to clusters exhibiting increased connectivity in relation to social support levels, we observe distinct patterns that further elucidate the left FP’s role in the neural network. The left FP’s increased connectivity with the right superior frontal gyrus (SFG) in individuals with high social support (β = 0.169, p = 0.0005) aligns with the SFG’s established functions in executive control and emotional regulation (89, 90). This enhancement potentially reflects the SFG’s and FP’s collaborative roles in higher cognitive processes and decision-making, underpinned by a supportive social milieu.

Moreover, the left FP’s interaction with the lateral occipital cortex’s superior division left shows a significant negative correlation in the low social support group (β = -0.180, p = 0.0057), emphasizing the modulation of visual processing networks by social support. Conversely, the high social support group maintains stable connectivity in this region, highlighting the protective effects of social support against connectivity reductions in critical visual processing areas.

Additionally, the enhanced connectivity between the left FP and regions including the lateral occipital cortex, superior division right, and angular gyrus right, in contexts of high social support, showcases the complex interplay between social support levels and neural connectivity across diverse cognitive and sensory domains. Specifically, the analysis demonstrated a significant negative correlation in the low social support group for this connectivity (β = -0.189, p = 0.0050), emphasizing the modulatory influence of social support. Conversely, in the high social support group, there is a non-significant positive trend, suggesting that high social support may mitigate or even reverse the negative impact on connectivity observed under lower social support conditions.

These findings elucidate a multifaceted neural interplay where social support emerges as a key modulator of connectivity patterns, underscoring its significance in shaping the brain’s adaptive mechanisms. The left FP, through its intricate connections moderated by social support, exemplifies the dynamic interplay between individual cognitive-emotional capabilities and their interaction with the social environment, fostering resilience and adaptive cognitive strategies as supported by the literature (16, 91–94).

The PCC emerged as the third region where functional connectivity is significantly influenced by social support, particularly in its connectivity with a cluster encompassing the left cerebellum (Crus2 Left and Crus1 Left).

Positioned at the core of the Default Mode Network (DMN) (95), the PCC plays a pivotal role in internal thought processes, memory recall, future planning, and mind-wandering (96–99), while also helping to mediate between internal and external stimuli (100, 101).

Our findings provided a nuanced perspective on how social support levels distinctly moderate the connectivity between the PCC and left cerebellar regions, Crus 1 and Crus 2. **Figure 8** illustrates a significant negative moderation effect on this connectivity in participants with low social support (β = -0.140, p = 0.0042), contrasting with a positive association in those with high social support (β = 0.065, p = 0.049). This differential influence highlights the role of social support in moderating the neural communication between the PCC and cerebellum, expanding our understanding of their involvement in higher-order cognitive and emotional functions beyond traditional sensorimotor coordination (102–104).

Recent research underscores the cerebellum’s contribution to non-motor functions, including emotional regulation and social cognition (105–107), aligning with its connectivity to the PCC. This moderation effect elucidates the cerebellum’s integral role in the social and emotional dimensions of brain function, as evidenced by its connectivity with the PCC being sensitive to the levels of perceived social support.

In sum, our findings enrich the discourse on the neural mechanisms underpinning resilience and the perception of social support, shedding light on the PCC and cerebellum’s coordinated roles in this context. By demonstrating how social support levels significantly moderate PCC-cerebellar connectivity, our research contributes to a more comprehensive understanding of the cerebellum’s extended roles within the DMN, emphasizing its relevance in the neural networks that underpin social cognition and emotional regulation in relation to resilience and social support (104, 107, 108).

The last seed region scrutinized in our study is the left hippocampus, which demonstrated a nuanced relationship with the paracingulate gyrus, influenced by the levels of social support. Known for its roles in memory consolidation, spatial navigation, and emotion regulation, the hippocampus is also pivotal in orchestrating complex cognitive tasks and modulating behavior in response to novel situations—capabilities central to resilience (109, 110). Additionally, its consistent association with resilience mechanisms is well documented (111).

Montagrin et al, (112), emphasize the hippocampus’s involvement in processing social information. Highlighting its support for social memory and the organization of social space, crucial for dynamic social interactions and the adaptation of social knowledge. Correspondingly, the paracingulate gyrus plays a significant role in decision-making, error monitoring (113), and social cognition (114), positioning it as a complementary neural hub in the hippocampal network.

The interaction analysis for the left hippocampus, depicted in **Figure 9**, indicated increased connectivity with the left paracingulate gyrus. However, this increase did not significantly vary with social support levels, showing non-significant trends for both low (β = 0.054, p = 0.120) and high social support groups (β = 0.023, p = 0.593). This outcome prompts further exploration into potential underlying factors, such as genetic variances or individual life experiences, that might modulate this relationship beyond the detected trends in our study. While our findings did not demonstrate a significant interaction effect, they suggest a trajectory for future research. The trend toward increased connectivity, though not statistically significant in our sample, may indicate a latent pattern that larger or longitudinal studies could uncover, offering deeper insights into the individual differences influencing the connectivity between these essential brain regions (115, 116). Such investigations could elucidate the complex interplay between the hippocampus and paracingulate gyrus, enriching our understanding of how these regions support cognitive and emotional integration within varied social contexts.

In our exploration, we delved into the sophisticated interplay between social support and resilience, anchoring our hypothesis that elevated levels of social support intensify the positive influence of resilience on brain connectivity, especially within areas critical to emotion regulation, cognitive processing, and adaptive problem-solving. The results from our study substantiate this hypothesis, illustrating that heightened social support modifies functional connectivity in regions essential for the brain’s adaptive mechanisms, as revealed through our pioneering use of ultra-high field resting-state fMRI.

Utilizing seed-based analysis with UHF resting-state fMRI, we identified that high social support significantly alters connectivity patterns in the left and right FP, the PCC, and the left hippocampus. Notably, the FPs and PCC—areas integral to emotion regulation and socio-cognitive processing—demonstrated connectivity changes that corroborate our hypothesis, suggesting that supportive social environments bolster the neural foundations of resilience.

While our study did not find the hypothesized interaction effect in the hippocampus’s connectivity with the paracingulate gyrus, the significant findings in other regions affirm the synergy between social support and resilience. This is particularly evident in the PCC’s interaction with the cerebellum, highlighting its role in emotional regulation and social cognition, pertinent to our understanding of resilience (95, 97).

The relevance of these findings extends to the context of mild traumatic brain injury, TBI and PTSD, where disruptions in emotional regulation, cognitive processing, and problem-solving are prevalent. Studies like those by Vasterling et al, (117) underscore the impact of social support in mitigating cognitive and emotional challenges associated with trauma, resonating with our results that advocate for the therapeutic potential of bolstering social networks to enhance resilience and aid recovery in these conditions.

Moreover, our insights align with digital mental health interventions, such as the FRIEND and Balsam apps, which operationalize the concept of social support to alleviate psychological distress (118, 119). Such tools exemplify how our findings can be translated into practical applications, offering personalized support and aligning with the therapeutic strategies for TBI and PTSD as discussed by Ozbay et al. (12) and reflected in the broader literature on social support and mental health.

In conclusion, our research affirms the hypothesis that high social support can amplify the brain’s resilience mechanisms, offering valuable perspectives on the neural interplay underlying these effects, captured through the use of UHF resting-state fMRI. This contribution is particularly salient for individuals with mild TBI and PTSD, where enhanced social support could play a critical role in recovery and rehabilitation. Moving forward, the implications of our findings beckon further inquiry into targeted interventions that leverage social support to foster resilience, providing new pathways to support recovery in individuals facing TBI, PTSD, and related stressors.

### 4.1 Limitations

In interpreting the findings of this study, several limitations warrant consideration. Firstly, the relatively small sample size employed in our research might constrain the external validity of our results, possibly not reflecting the diversity and nuances of larger populations. Consequently, replicating this study with a larger and more diverse sample size becomes a pivotal step in accentuating and affirming the robustness of our observations.

## Data availability statement

The raw data supporting the conclusions of this article will be made available by the corresponding author, Prof. Dr. Irene Neuner, without undue reservation.

## Ethics statement

The studies involving humans were approved by The Ethics Committee of the Medical Faculty of RWTH Aachen University, and all participants provided written informed consent. The studies were conducted in accordance with the local legislation and institutional requirements. The participants provided their written informed consent to participate in this study.

## Author contributions

NK: Writing – review & editing, Writing – original draft, Methodology, Investigation, Formal analysis, Conceptualization. SR: Writing – review & editing, Software, Methodology, Formal analysis, Data curation, Conceptualization. DN: Writing – review & editing, Data curation, Investigation. DA: Writing – review & editing, Data curation. RR: Writing – review & editing, Methodology, Formal analysis, Data curation. JD: Writing – review & editing, Formal analysis. JS: Writing – review & editing, Supervision, Resources, Funding acquisition. TV: Writing – review & editing, Validation, Supervision, Methodology, Conceptualization. IN: Writing – review & editing, Validation, Supervision, Resources, Project administration, Methodology, Funding acquisition, Conceptualization.
