# Supplementary material for: The interaction effect of high social support and resilience on functional connectivity using seed-based resting-state assessed by 7-Tesla ultra-high field MRI
Source: Front Psychiatry. 2024 May 20;15:1293514. doi: 10.3389/fpsyt.2024.1293514 (PMC11145276; doi:10.3389/fpsyt.2024.1293514)
Supplement: Supplementary file 1 [file DataSheet_1.zip › Table 1.docx]

Supplementary Material

Table 1: Bonferroni Correction applied to multiple comparisons across eight seed regions in seed-to-voxel analysis.

| Seed Region | Uncorrected P-value | Significance After Bonferroni Correction |
| --- | --- | --- |
| FP-r | 0.000063 | Significant |
| FP-l Cluster 1 | 0.000125 | Significant |
| FP-l Cluster 2 | 0.000028 | Significant |
| FP-l Cluster 3 | 0.000004 | Significant |
| FP-l Cluster 4 | 0.000190 | Significant |
| PCC | 0.000000 | Significant |
| Hippocampus | 0.000011 | Significant |
| Bonferroni-corrected Alpha Threshold | 0.006250 |  |
