# Supplementary material for: The interaction effect of high social support and resilience on functional connectivity using seed-based resting-state assessed by 7-Tesla ultra-high field MRI
Source: Front Psychiatry. 2024 May 20;15:1293514. doi: 10.3389/fpsyt.2024.1293514 (PMC11145276; doi:10.3389/fpsyt.2024.1293514)
Supplement: Supplementary file 1 [file DataSheet_1.zip › Table 2.DOCX]

Supplementary Material

Table 2: Sensitivity Analysis Results (Moderation Analysis). MNI coordinates (x, y, z) represent peaks within a cluster. Cluster size corresponds to the spatial extent (i.e., volume (mm3)). Multiple comparisons were corrected using family-wise error correction at the cluster level.

| Region of interest | Cluster # | MNI coordinates (x,y,z) | Cluster size (mm^3^) | Brain regions | p-unc | p-FDR | T-value | Effect size |
| --- | --- | --- | --- | --- | --- | --- | --- | --- |
| FP-r | 1 | -42 -64 +42 | 1,792 | Lateral Occipital Cortex, superior division Left  Angular Gyrus Left | 0.000094 | 0.000094 | 4.72 | 0.24 |
| FP-l | 3 | -58 -32 +14  +12 +38 +44  -40 -72 +46 | 808  856  808 | Planum Temporale Left  Parietal Operculum Cortex Left  Superior Frontal Gyrus Right  Frontal Pole Right  Lateral Occipital Cortex, superior division Left | 0.000165  0.000035  0.000009 | 0.000165  0.000052  0.000027 | -4.49  5.12  5.68 | -0.22  0.21  0.19 |
| PCC | 1 | -36 -74 -36 | 3,016 | Cerebellum Crus2 Left  Cerebellum Crus1 Left | 0.000001 | 0.000001 | 6.87 | 0.17 |
| Hippocampus-l | 1 | -08 +44 +22 | 880 | Paracingulate Gyrus Left | 0.000010 | 0.000010 | 5.61 | 0.15 |

Table 3: Seed-based functional connectivity analysis results (Resilience main effect). MNI coordinates (x, y, z) represent peaks within a cluster. Cluster size corresponds to the spatial extent (i.e., volume (mm3)). Multiple comparisons were corrected using family-wise error correction at the cluster level.

| Region of interest | Cluster # | MNI coordinates (x,y,z) | Cluster size (mm3) | Brain regions | | p-unc | p-FDR | T-value | Effect size |
| --- | --- | --- | --- | --- | --- | --- | --- | --- | --- |
| FP-r | 3 | -22 -72 -22  +34 -50 -18  +34 -60 +38 | 9280  1528  1384 | | Cerebellum 6 Left  Cerebellum Crus 1 Left  Cerebellum Crus 1 Left  Temporal Occipital Fusiform Cortex Left  Cerebellum 45 Left  Cerebellum 8 Left  Temporal Occipital Fusiform Cortex Right  Cerebellum 6 Right  Cerebellum Crus 1 Right  Lateral Occipital Cortex, superior division Right | 0.000000  0.000024  0.000026 | 0.000000  0.000026  0.000026 | 7.71  5.22  -5.19 | 0.18  0.16  -0.24 |
| ACC | 1 | +36 +08 +50 | 3,160 | | Middle Frontal Gyrus Right | 0.000001 | 0.000001 | -6.72 | -0.19 |

Table 4: Seed-based functional connectivity analysis results (Social support main effect). MNI coordinates (x, y, z) represent peaks within a cluster. Cluster size corresponds to the spatial extent (i.e., volume (mm3)). Multiple comparisons were corrected using family-wise error correction at the cluster level.

| Region of interest | Cluster # | MNI coordinates (x,y,z) | Cluster size (mm^3^) | Brain regions | | p-unc | p-FDR | T-value | Effect size |
| --- | --- | --- | --- | --- | --- | --- | --- | --- | --- |
| FP-r | 2 | -24 -70 -22  -44 -64 +46 | 2240  936 | | Cerebellum 6 Left  Cerebellum Crus 1 Left  Occipital Fusiform Gyrus Left  Lateral Occipital Cortex, superior division Right | 0.000000  0.000046 | 0.000001  0.000046 | -7.00  4.96 | -0.15  0.21 |
| ACC | 2 | +34 +10 +58  +46 -54 +52 | 2496  1616 | | Middle Frontal Gyrus Right  Angular Gyrus Right  Lateral Occipital Cortex superior division Right  Superior Parietal Lobule Right | 0.000001  0.000007 | 0.000002  0.000007 | 6.43  5.69 | 0.18    0.19 |
| PCC | 1 | -08 -78 -40 | 2040 | | Cerebellum Crus2 Left  Cerebellum 7b Left  Vermis 8  Cerebellum 8 Left | 0.000009 | 0.000009 | 5.62 | 0.18 |
| Hippocampus-r | 1 | -12 +44 +40 | 1768 | | Frontal Pole Left  Superior Frontal Gyrus Left | 0.000000 | 0.000000 | 7.01 | 0.16 |
