# Supplementary material for: The interaction effect of high social support and resilience on functional connectivity using seed-based resting-state assessed by 7-Tesla ultra-high field MRI
Source: Front Psychiatry. 2024 May 20;15:1293514. doi: 10.3389/fpsyt.2024.1293514 (PMC11145276; doi:10.3389/fpsyt.2024.1293514)
Supplement: Supplementary file 1 [file DataSheet_1.zip › Table 3.docx]

Supplementary Material

Table 3: Sensitivity Analysis Results (Moderation Analysis). MNI coordinates (x, y, z) represent peaks within a cluster. Cluster size corresponds to the spatial extent (i.e., volume (mm3)). Multiple comparisons were corrected using family-wise error correction at the cluster level.

| Region of interest | Cluster # | MNI coordinates (x,y,z) | Cluster size (mm^3^) | Brain regions | p-unc | p-FDR | T-value | Effect size |
| --- | --- | --- | --- | --- | --- | --- | --- | --- |
| FP-r | 1 | -42 -64 +42 | 1,792 | Lateral Occipital Cortex, superior division Left  Angular Gyrus Left | 0.000094 | 0.000094 | 4.72 | 0.24 |
| FP-l | 3 | -58 -32 +14  +12 +38 +44  -40 -72 +46 | 808  856  808 | Planum Temporale Left  Parietal Operculum Cortex Left  Superior Frontal Gyrus Right  Frontal Pole Right  Lateral Occipital Cortex, superior division Left | 0.000165  0.000035  0.000009 | 0.000165  0.000052  0.000027 | -4.49  5.12  5.68 | -0.22  0.21  0.19 |
| PCC | 1 | -36 -74 -36 | 3,016 | Cerebellum Crus2 Left  Cerebellum Crus1 Left | 0.000001 | 0.000001 | 6.87 | 0.17 |
| Hippocampus-l | 1 | -08 +44 +22 | 880 | Paracingulate Gyrus Left | 0.000010 | 0.000010 | 5.61 | 0.15 |
