# Supplementary material for: The interaction effect of high social support and resilience on functional connectivity using seed-based resting-state assessed by 7-Tesla ultra-high field MRI
Source: Front Psychiatry. 2024 May 20;15:1293514. doi: 10.3389/fpsyt.2024.1293514 (PMC11145276; doi:10.3389/fpsyt.2024.1293514)
Supplement: Supplementary file 1 [file DataSheet_1.zip › Table 4.docx]

Supplementary Material

Table 4: Seed-based functional connectivity analysis results (Resilience main effect). MNI coordinates (x, y, z) represent peaks within a cluster. Cluster size corresponds to the spatial extent (i.e., volume (mm3)). Multiple comparisons were corrected using family-wise error correction at the cluster level.

| Region of interest | Cluster # | MNI coordinates (x,y,z) | Cluster size (mm3) | Brain regions | | p-unc | p-FDR | T-value | Effect size |
| --- | --- | --- | --- | --- | --- | --- | --- | --- | --- |
| FP-r | 3 | -22 -72 -22  +34 -50 -18  +34 -60 +38 | 9280  1528  1384 | | Cerebellum 6 Left  Cerebellum Crus 1 Left  Cerebellum Crus 1 Left  Temporal Occipital Fusiform Cortex Left  Cerebellum 45 Left  Cerebellum 8 Left  Temporal Occipital Fusiform Cortex Right  Cerebellum 6 Right  Cerebellum Crus 1 Right  Lateral Occipital Cortex, superior division Right | 0.000000  0.000024  0.000026 | 0.000000  0.000026  0.000026 | 7.71  5.22  -5.19 | 0.18  0.16  -0.24 |
| ACC | 1 | +36 +08 +50 | 3,160 | | Middle Frontal Gyrus Right | 0.000001 | 0.000001 | -6.72 | -0.19 |
