# Supplementary material for: The interaction effect of high social support and resilience on functional connectivity using seed-based resting-state assessed by 7-Tesla ultra-high field MRI
Source: Front Psychiatry. 2024 May 20;15:1293514. doi: 10.3389/fpsyt.2024.1293514 (PMC11145276; doi:10.3389/fpsyt.2024.1293514)
Supplement: Supplementary file 1 [file DataSheet_1.zip › Table 5.docx]

Supplementary Material

Table 5: Seed-based functional connectivity analysis results (Social support main effect). MNI coordinates (x, y, z) represent peaks within a cluster. Cluster size corresponds to the spatial extent (i.e., volume (mm3)). Multiple comparisons were corrected using family-wise error correction at the cluster level.

| Region of interest | Cluster # | MNI coordinates (x,y,z) | Cluster size (mm^3^) | Brain regions | | p-unc | p-FDR | T-value | Effect size |
| --- | --- | --- | --- | --- | --- | --- | --- | --- | --- |
| FP-r | 2 | -24 -70 -22  -44 -64 +46 | 2240  936 | | Cerebellum 6 Left  Cerebellum Crus 1 Left  Occipital Fusiform Gyrus Left  Lateral Occipital Cortex, superior division Right | 0.000000  0.000046 | 0.000001  0.000046 | -7.00  4.96 | -0.15  0.21 |
| ACC | 2 | +34 +10 +58  +46 -54 +52 | 2496  1616 | | Middle Frontal Gyrus Right  Angular Gyrus Right  Lateral Occipital Cortex superior division Right  Superior Parietal Lobule Right | 0.000001  0.000007 | 0.000002  0.000007 | 6.43  5.69 | 0.18    0.19 |
| PCC | 1 | -08 -78 -40 | 2040 | | Cerebellum Crus2 Left  Cerebellum 7b Left  Vermis 8  Cerebellum 8 Left | 0.000009 | 0.000009 | 5.62 | 0.18 |
| Hippocampus-r | 1 | -12 +44 +40 | 1768 | | Frontal Pole Left  Superior Frontal Gyrus Left | 0.000000 | 0.000000 | 7.01 | 0.16 |
